# Structure and Dynamics of Reentrant Nematics: Any Open Questions after Almost 40 Years?

**DOI:** 10.3390/ijms12085352

**Published:** 2011-08-22

**Authors:** Marco G. Mazza, Martin Schoen

**Affiliations:** 1 Stranski Lab for Physical and Theoretical Chemistry, Berlin Institute of Technology, 135-17 Juni Street, Berlin 10623, Germany; 2 Department of Chemical and Biomolecular Engineering, North Carolina State University, 911 Partners Way, Raleigh, NC 27695, USA

**Keywords:** reentrant phase, nematic, dynamics, diffusion

## Abstract

Liquid crystals have attracted enormous interest because of the variety of their phases and richness of their application. The interplay of general physical symmetries and specific molecular features generates a myriad of different phenomena. A surprising behavior of liquid crystals is the reentrancy of phases as temperature, pressure, or concentration are varied. Here, we review the main experimental facts and the different theoretical scenarios that have guided the understanding of bulk reentrant nematics. Recently, some computer simulations of a system confined to nanoscopic scales have found new dynamical features of the reentrant nematic phase. We discuss this prediction in relation with the available experimental evidence on reentrant nematics and with the dynamics of liquids in strongly confined environments.

## 1. Introduction

**Figure 1.**
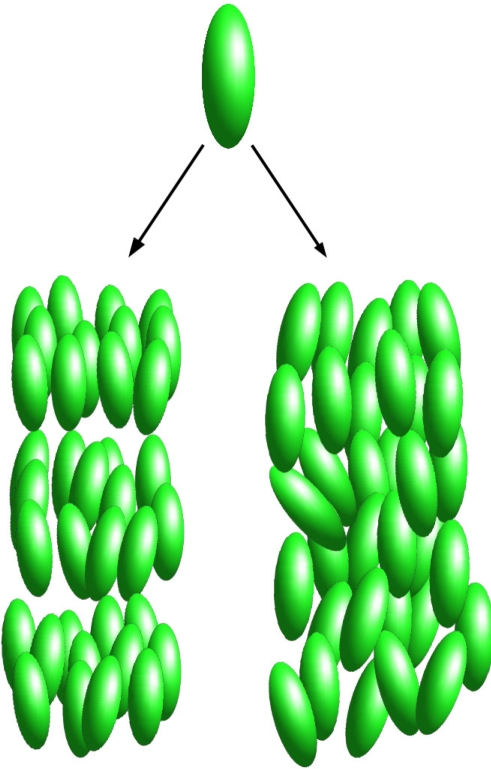
Two examples of mesophases formed by rod-like molecules. Smectic A (bottom left) and nematic (bottom right).

**Figure 2.**
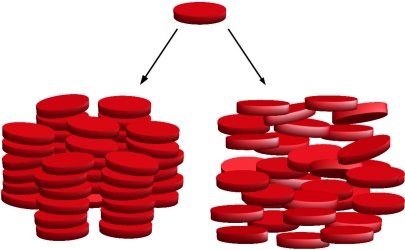
Two examples of mesophases formed by disk-like molecules. Columnar (bottom left) and nematic discotic (bottom right).

In 1888 the Austrian chemist and botanist Friedrich Reinitzer observed that the organic compound cholesteryl benzoate exhibits two melting points. He found that it first melts at 145.5 °C to a cloudy liquid, and then at 178.5 °C it becomes suddenly clear. Later, the pioneering studies of Lehmann [1], Schenk [2], Vorländer [3] and Friedel [4] recognized a new state of matter intermediate between liquid and solid. Liquid crystals (LC’s) were discovered. We now know a multitude of different liquid crystalline phases, roughly divided into two families. (i) *Thermotropic* LC’s undergo phase transitions as the temperature (*T*) is changed; (ii) *Lyotropic* LC’s exhibit different phases as the solvent concentration is changed. The most commonly encountered phases are *nematic* (N), where the system has a preferential orientation given by the nematic director *n̂*, but the centers of mass have no long-range correlation (see the bottom right panel of Figure 1); *smectic* (S), where molecules organize in layers (see the bottom left panel of Figure 1); *columnar*, where two-dimensional order is established (see Figure 2); *cholesteric*, where the average orientation spirals with a fixed pitch as different planes are traversed, and *blue phases*, which consist of three-dimensional arrangements of cholesteric tubes.

Many different molecules form liquid crystalline phases, and their modern importance is difficult to exaggerate. Liquid crystal displays for electronic devices are ubiquitous. Because of the anisotropy of LC molecules and of their interactions, LC’s can easily polarize light passing through them. Highly versatile lasers can be made with LC systems, promising numerous applications [5]. Liquid crystals are extremely important also for biology. Some examples (far from being comprehensive) are the following: The lipid bilayer of a cell membrane is a stable LC phase on which depends the very existence of cells; strands of DNA are also known to form LC’s [6,7]; spider’s silk has exceptional materials properties due to the particular LC organization of its proteins [8]; finally, LC’s are ushering a technological revolution with multiple applications in biology and medicine as biosensors [9]. Many extensive reviews of the state of the art of LC crystals exist, see for example [10] for modern applications of LC in self-assembling supramolecular structures.

Among the interesting features of LC’s that make them so versatile there is a surprising sequence of phase transitions of rod-like molecules that still poses challenges to both experimental and theoretical studies. The typical sequence of LC phases encountered starts at high *T* with an isotropic (I) phase, where the system is characterized by complete translational and orientational invariance. As *T* decreases a preferential molecular orientation emerges—the nematic director—breaking the rotational symmetry. At even lower *T*, S layers appear indicating that now translational invariance is broken, too.

Surprisingly, in 1975 Cladis discovered [11] that when two compounds, whose molecules have two benzene rings and a strongly polar cyano group, were mixed together, a N phase formed at *T above* the S phase (which is usual), but also at *T below* the S phase (which is surprising). The N phase at lower *T* was termed *reentrant nematic* (RN). The presence of a RN phase is surprising because in the vast majority of cases as *T* is lowered the symmetry of the stable thermodynamic phase decreases, *i.e.*, the state of the system is invariant under a decreasing number of symmetry operations, eventually becoming a crystalline solid.

After the seminal work of Cladis [11], other systems in a variety of different conditions were found to have a RN phase. Today, RN behavior is a well-established phenomenon. Here, we focus on the past and present understanding of RN, with special attention to the dynamics of RN phases.

Naturally, nearly 40 years after Cladis’ discovery of the RN phase one may wonder: Is there still something to be understood about these phases? It is the primary purpose of this article to demonstrate that it is indeed so. For example, recent numerical investigations on the dynamics of LC’s confined in nanoscopic pores suggest some interesting dynamics features. In particular, it was found [12,13] that diffusivity in the direction of the molecular long axis is greatly enhanced. This appears to be an analogous situation to the levitation effect in zeolites and carbon nanotubes [14,15]. There is some favorable experimental evidence [16] supporting the theoretical predictions of enhanced parallel diffusivity [12,13]. However, the question is not yet settled. Our critical review of the available experimental work on RN dynamics reveals that numerous controversies are not yet resolved. A main problem identified here is that reentrancy is not caused by a single microscopic mechanism, but rather by many. Their identification is still an open question. Consequently, the investigations of the dynamical behavior of RN’s are often divergent.

The remainder of this Review is organized as follows: in Section 2 we review the seminal experiments that discovered RN phases in both polar and nonpolar substances. In Section 3 we discuss the main experimental investigations on the nature and properties of RN’s, while in Section 4 we review the different theories and scenarios put forward to rationalize RN existence and features. In Section 5 we review the more recent computational studies of RN’s, and, finally, Section 6 lists our conclusions.

## 2. History

The first experimental detection of RN in a LC is reported by Cladis [11]. There, an amount of hexyloxybenzilidene amino benzonitrile (HBAB) was mixed with cyanobenzilidene butylaniline (CBOOA). Individually, the former has a N phase for 35 °C ≲ *T* ≲ 101 °C, and the latter has a S_A_ phase for 44 °C ≲ *T* ≲ 83 °C and a N phase for 83 °C ≲ *T* ≲ 108 °C. When mixed together, as *T* is lowered a sequence of I, N, S_A_, and RN is found for a range of molar fractions *c*; the curve bounding the S_A_ phase in the *T* − *c* plane can be approximately fitted with a parabola

(1) $$c=c_{0}+\beta {\left(T_{\text{NS}}-T_{0}\right)}^{2}$$

where *c*_0_ ≈ 0.09, *β* ≈ − 5.4 × 10^3^ °C^−2^, *T*_0_ ≈ 61 °C, and *T*_NS_ is the N-S_A_ transition *T* [11]. The measurements of the bend elastic constant [11] constitutes the first examination of possible structural differences between the N and RN phases. In this early experiment it was shown that the elastic constant has a very similar *T* -behavior as the S_A_ phase is approached from above or below [11]. Cladis [11] emphasized that CBOOA has an incommensurate S_A_ phase, that is, the spacing between S layers is not an integral multiple of the molecular length. A possible way to understand how an incommensurate spacing can arise is to imagine a S layer composed of dimers. Because of their strongly polar character, the molecules of cyano compounds naturally tend to form dimers to minimize the interaction energy; these dimers in turn organize themselves in S layers, or, in other words, each S layer is in reality a double layer of molecules. This initial observation is consistent with other experiments [17] and has proven itself fruitful in the understanding of the structure of RN phases.

Following the assumption of the double-layer nature of the S_A_ phase, a series of pure compounds and some mixtures were studied [18]. Upon increasing pressure, RN phases were found in the supercooled region of polar substances such as 4-cyano-4′-octyloxy biphenyl (COOB) or CBOOA. What these substances have in common is the amphiphilic nature of their molecules. Unlike most LC’s, the polar part (the aromatic rings) is not in the middle of the chains, but rather at one end. The polar cyano group has a dipole moment of 4.5 D. The nonpolar part (aliphatic chain) forms the opposite end of the amphiphile. This structure will naturally favor a certain degree of dimerization, since the polar groups will preferentially interact with each other through long-range forces, and the nonpolar tails through short-range forces. Cladis *et al.* [18] proposed that it is the short-range interaction between nonpolar tails what stabilizes the S layers. This hypothesis is successfully tested by measuring the dependence of the maximum stability pressure *P_m_* of the S_A_ on the number of carbon atoms in the nonpolar tails. It is found that *P_m_* increases linearly as the number of methylene groups increases. It is argued [18,19] that as pressure increases (or equivalently as *T* decreases) the interaction between the polar groups becomes repulsive, and at the same time the nonpolar tails are somewhat compressed, lowering the energy barrier to permeation through the S layers. Both effects lead to a destabilization of the S order, and hence to the formation of a RN.

After 1979, the occurrence of RN could not be dismissed as a peculiarity of few molecules, since a stable RN at atmospheric pressure was found for molecules exhibiting three benzene rings, and, importantly, not in mixtures but in pure compounds [20–22]. Additionally, reentrant *smectic* phases were also found [23,24]. In the following years different groups found many different systems exhibiting RN phases (see, e.g., the work of Sigaud *et al.* [22] and references therein), and also multiple reentrant phases [25,26]. It has been speculated that quadruple reentrance is possible if compounds with four or five benzene rings are employed [27]. More details on the discovery and thermodynamic understanding of RN phases provides many more details [28].

Until 1983 it was believed that only molecules with a strong polar group showed a reentrant behavior. However, Diele *et al.* showed the existence of RN phases in terminal-nonpolar substances [29,30]. Importantly, their X-ray measurements showed that the thickness of the S layers is equal to the average molecular length. Hence, dimerization cannot take place in these nonpolar compounds and the occurrence of a RN phase must have a different physical origin [29,30] (see Section 4 for the theories applicable to nonpolar systems).

A question naturally arising is whether RN and N phases are different. One can consider two aspects: structure and dynamics. In the following we explore the experimental evidence addressing this question. We can, however, anticipate that, since pure compounds and mixtures, polar and nonpolar molecules all exhibit RN phases, the picture emerging will be rather complex and diverse.

## 3. Experimental Investigations

Structural differences of the N and RN phases were investigated with X-ray scattering [31] for mixtures of N-(*p*-hexyloxybenzylidene)-*p*-aminobenzonitrile (HBAB) and N-*p*-cyanobenzylidene-*p*-*n-*octyloxyaniline. No qualitative difference was found between the N and RN phases, *i.e.*, the Bragg peak indicating the layer thickness appears always in the range 33–35Å; however, for some mixtures the RN was found to coexist with microcrystallites. Guillon *et al.* concluded that these crystalline fluctuations share the same structural properties and are energetically similar to the RN phase [31].

Nuclear magnetic resonance (NMR) offers a number of techniques to investigate both structure and dynamics. The first NMR study of a pure LC system with a RN phase was carried out by Miyajima *et al.* [32]. For pure 4-(4″-octyloxybenzoloxy)-benzylidene-4′-cyanoaniline (OBBC) they found a smooth continuous increase in the orientational order parameter across the N, S_A_, and RN phases. However, when studying a binary mixture with a spin-probe impurity probe they found a steep increase of the nematic order parameter in the RN phase. While proton NMR is sensitive to the aromatic core, the impurity probe method is not. Therefore, the conflicting results in the orientational order parameter can be ascribed to the different techniques. From the *T* behavior of the spin-lattice relaxation time *T*_1_ measured at 29.8 MHz for OBBC Miyajima *et al.* conclude that translational self-diffusion plays a dominant role in *T*_1_ relaxation in the RN phase (with a characteristic Arrhenius *T*-dependence), while the N phase *T*_1_ behavior is dominated by nematic director fluctuations.

However, for a LC mixture Dong reached the opposite conclusion that self-diffusion appears to be the relevant relaxation mechanism in the N phase, while nematic director fluctuations dominate the RN phase [33–35]. Using proton NMR he studied the *T* dependence of *T*_1_ for a LC binary mixture [33,34] and found an Arrhenius behavior in the N phase, and no *T*-dependence in the RN phase within experimental error; that director fluctuations dominate the relaxation of the RN phase is further corroborated by the linear relationship between $T_{1}^{-1}$ and *ω*^−1/2^ where *ω* is the angular frequency, as predicted by the theory of spin relaxation through a director-fluctuation mechanism [35].

We cannot avoid stressing at this point something that has not been clearly stated in previous works. Although reentrancy is found in many different substances, their dynamics need not be the same. Specifically, pure compounds and mixtures do not necessarily have to exhibit the same dynamical properties. Even though there are discrepancies in the experimental results, we can safely conclude that the dynamics of N and RN are markedly different.

A somewhat intermediate situation is described by Sebastião *et al.* [36] who performed proton NMR experiments on a polar LC compound which exhibits N, partial bilayer S, RN, and reentrant S_A_ phases. Self-diffusion is shown to contribute little to *T*_1_ in the N and RN phase. Instead, fluctuations of the director and reorientations are the main dynamical processes contributing to *T*_1_. Interestingly, however, the ratio between the translational self-diffusion parallel and perpendicular to the director is slightly higher in the RN phase than in the N phase (see Table 1 in [36]). The results of [36] are consistent with a dynamical process of association and dissociation of molecular groups, which are important in the double-layer S and N phases.

By using ^129^Xe NMR, Bharatan and Bowers [37] studied two LC mixtures and found that *T*_1_ (and also the spin-spin relaxation time *T*_2_) shows Arrhenius behavior in both N and RN phases. The activation energies extracted from the data do not behave in a similar fashion for the two systems studied. For a binary mixture, the activation energy in the RN is more than two times the value in the N phase, whereas for a ternary mixture, there is almost no difference in the activation energies. The increase in activation energy for the first LC mixture is ascribed to changes in the molecular packing in the RN phase [37].

Although a number of other studies were carried out on mixtures and pure compounds with a RN phase [38–40] using deuteron NMR, little can be said about translational self-diffusion, because this technique is sensitive to other types of motion, e.g., intramolecular rotations, reorientational dynamics, or supramolecular motion, such as order director fluctuations. The picture arising from the work of Dong and collaborators is one of very subtle changes between the phases, and especially between N and RN phases.

The structure of RN’s also poses some unanswered questions. For example, is there a change in nematic order parameter *Q* at the transition from S to RN? The work of Vaz *et al.* [41] shows that *Q* increases upon entering the RN. They measured the *T*-dependence of *Q* in a LC binary mixture with deuterium NMR and found that at the S_A_-RN transition *Q* displays a sharp increase in slope [41]. To rationalize this behavior they considered an expansion of the free energy difference Δ*F* ≡ *F_N_* +*F_S_* −*F_I_* between the N, S and I phase in terms of *Q* and a smectic order parameter Ψ, that to leading order is

(2) $$\Delta F=\frac{1}{2}\alpha Q^{2}+\frac{1}{3}\beta Q^{3}+\frac{1}{4}\gamma Q^{4}+\frac{1}{2}a\Psi^{2}+\frac{1}{4}b\Psi^{4}$$

where *α* = *α*_0_(*T* − *T_IN_*), *a* = *a*_0_(*T* − *T_NA_*), *T_IN_* and *T_NA_* are the I-N and the N to S_A_ transition *T*, respectively, and all the constants are positive except *β* < 0. This expansion of Δ*F* produces a first order I to N phase transition, and a second order N to S_A_ transition. Following Cladis [42], they assumed a quadratic coupling of the order parameters proportional to *Q*^2^Ψ^2^ to describe the RN phase and derived that upon entering the RN from the S_A_ phase the nematic order parameter should be positively perturbed (*δQ* > 0) such that the phenomenological description is in agreement with the experiments [41].

However, using deuterium NMR on a binary mixture, Emsley *et al.* [43] found only subtle changes in the order parameters at the S_A_-RN phase transition. Contrasting results are also presented by Dong *et al.* [44], where deuterium NMR experiments showed no enhanced orientational order in the RN phase, in contradiction with both a Landau free energy expansion theory and a McMillan theory of RN’s.

Electron spin resonance experiments on the LC mixture of hexyloxy biphenyl (6OCB) and octyloxy biphenyl (8OCB) found no dramatic structural changes at the S_A_-RN transition using a number of different spin probe molecules and similar dynamics of the probes in reentrant and nonreentrant LC [45]. Nayeem and Freed also concluded, contrary to Dong *et al.* [38], that their results are “*not consistent with saturation in pair formation being a necessary precursor to reentrance*” [45]. The differences in these experimental results can be ascribed to different experimental techniques sensitive to a different extent to the orientational degrees of freedom of the aromatic cores or of the molecular chains. Nonetheless, the question still lingers: What is the behavior of the nematic order across the S-RN phase transition?

A very different experimental approach was adopted by Ratna *et al.* [16] who measured the electrical conductivity in the direction parallel (*σ*_‖_) and perpendicular (*σ*_⊥_) to the optic axis, which in the orientationally ordered phases coincides with the nematic director. They studied the pure compound 4-cyanophenyl-3′-methyl-4(4′-*n*-dodecylbenzoyloxy)benzoate (12 CPMBB) and the mixture 6OCB/8OCB. For the single component system they found a remarkable increase of the ratio *σ*_‖_/*σ*_⊥_ as the system approaches the RN-S_A_ transition temperature *T_RN_*_−_*_A_*. At the lowest *T* studied for the pure compound (1.2 °C below *T_RN_*_−A_) *σ*_‖_ is approximately equal to 16*σ*_⊥_. For the mixture 6OCB/8OCB, they found the same qualitative increase of the ratio *σ*_‖_/*σ*_⊥_, but at the lowest *T* studied in the RN phase the value of this ratio was only 1.8 [16]. Although the results for the mixture would require additional analysis, what was found for the pure compound is strong evidence supporting a scenario of increased mass transport in the RN phase.

Another insight into the differences between N and RN phases comes from the work of Nozaki *et al.* [46] who found that for the mixture 6OCB/8OCB the rotations around the short molecular axis are greatly hindered in the RN phase with respect to the N phase. Specifically, by measuring the *T*-dependence of the complex permittivity with time-domain reflectometry, they found a higher value of the activation energy in the RN compared with the N phase.

There are limited modern investigations of the RN state. Yethiraj *et al.* measured proton NMR spectra of solutes dispersed in a mixture of 6OCB/8OCB to determine the smectic and nematic order parameters in the Kobayashi-McMillan theory [47]. Das and Prasad [48] measured the rotational viscosity of a LC binary mixture and found similar values in the N and RN phases. However, there are strongly contrasting results [34,49] suggesting that the rotational viscosity of RN may be two or three orders of magnitude higher than in the N phase.

Finally, hydrodynamic flow in RN’s has received some attention. Bhattacharya and Letcher measured capillary shear flow for a ternary mixture with a RN phase. They found that the N and RN phases have identical flow properties, and the hydrodynamic theory of N phases can be applied to RN phases [49]. Also, Jadżyn and Czechowski [50] measured the viscosity for the well studied mixture 6OCB and 8OCB, and found a distinct decrease of viscosity upon entering the RN phase.

## 4. Theoretical Scenarios for Reentrancy

The experimental findings discussed in Section 3 well illustrate the complexity of RN phases. It is therefore not surprising that a similar rich picture also emerges from theoretical studies. Prost and Barois derived a Landau theory of LC exhibiting reentrancy from general considerations of symmetry and on the basis of the relevant length scales involved [51,52]. In a S phase a density modulation appears. On average the molecular centers lie on parallel planes. Thus, it is natural to consider the expansion of the density in a Fourier series

(3) $$\rho =\rho_{0}+\frac{1}{\sqrt{2}}\left(\psi e^{iq\cdot r}\right)+\ldots$$

where the wave-vector **q** = (2*π*/*l*)*n̂*, *l* is the layer thickness, and *n̂* is the director. However, polar substances and mixtures exhibiting RN phases show two incommensurate periodicities in X-ray scattering experiments, *i.e.*, one periodicity equal to the molecular length *l*, and a second one *l′* that is not an integer multiple of the first, typically *l′* ≈ 1.2–1.5 *l*. It is necessary to introduce the total dipole moment **P**(r) to describe a system of polar molecules

(4) $$P(r)\equiv \frac{1}{V}\sum_{i}p_{i}\delta \left(r-r_{i}\right)$$

where **p***_i_* is the dipole moment of molecule *i*. A potential can be derived from **P**(r) that may be used as a second order parameter

(5) $$P(r)=\frac{1}{4\pi }\nabla \phi$$

With these two order parameters, *ρ* and *φ*, a Landau–Ginzburg free energy functional can be written as

(6) $$F=\frac{1}{2}\int d^{3}r\{A_{\rho }\rho^{2}+A_{\phi }\phi^{2}-D\rho \phi^{2}+\frac{1}{2}B_{\rho }\rho^{4}+\frac{1}{2}B_{\phi }\phi^{4}+C\rho^{2}\phi^{2}\}$$

From the competition between the different length scales contained in Equation 6, Prost and collaborators [51,52] obtained a phase diagram with different S and also RN phases.

The first microscopic theory of the physical origin of RN phases was elaborated by Berker and Walker in 1981 [53]. They started from the observation that most reentrant nematogens are composed of molecules with a strong polar group at one end and an attached aliphatic chain forming the other end of the molecule. The dipolar interaction is assumed to be antiferroelectric. Considering a plane normal to the nematic director, antiferroelectric long-range order cannot be established in a periodic regular arrangement of molecules. However, Berker and Walker argued that in a liquid, molecules can relax their positional degrees of freedom, and configurations of triplets with two short antiferroelectric bonds and one unfavorable ferroelectric but longer bond will be possible. In this way frustration is avoided and the system can condense to a liquid of dimers forming a two-dimensional network. Hence, a bilayer S phase forms. As *T* is lowered positional order arises that leads to increasing frustration and to the breaking of the dimers. As a consequence a nematic phase appears again. The interaction of two dipoles s_1_ and s_2_ separated by a distance |r_12_| is described by the potential

(7) $$U(r_{1},s_{1},r_{2},s_{2})=\frac{As_{1}\cdot s_{2}-3B\left(s_{1}\cdot r_{12}\right)\left(s_{2}\cdot r_{12}\right)}{{\left|r_{12}\right|}^{3}}$$

where |s*_i_*| = 1. For *B* < *A* the interaction is antiferroelectric, whereas for *B* = *A* it is purely dipolar, and for *B* > *A* the interaction becomes ferroelectric. By integrating out the positional degrees of freedom and distinguishing different degrees of interaction, Berker and Walker mapped the initial system onto an Ising model with annealed bond disorder. The phase diagram obtained [53] shows a RN phase for a range of model parameters and also a doubly reentrant phase sequence. Berker *et al.* [27,54] extended their theory to describe multiple reentrant behavior, considering partial bilayer (S_Ad_) and monolayer (S_A1_) smectic phases, *i.e.*, N–S_Ad_–N–S_Ad_–N–S_A1_, which was found experimentally in at least one pure substance and its mixture [55] and S_C_ phases [56]. The scenario of frustrated dipolar interaction [27,54] (commonly referred to as “spin-gas theory”) has played a major role in understanding different instances of reentrancy in LC [28]. An implication of the scenario described above is that the N-S_A_ transition is different from the S_A_-RN transition. This is at odds with some experiments that show very subtle or no structural difference between the nematic phases for LC mixtures [45,57] and a pure compound [58].

A different approach to a microscopic theory for reentrancy in nematogens was proposed by Longa and de Jeu [59]: Pairs of molecules with antiparallel dipoles associate to form dimers in dynamical equilibrium with monomers. As *T* is decreased the fraction of dimers in the LC system increases and dipolar dispersive forces between the dimers lead to the formation of a S_A_ phase. It is important to note that monomers under these conditions help stabilizing the S_A_ phase because monomers can fill the voids left by the bulkier dimers. However, as the dimer fraction increases it becomes increasingly difficult to pack them without perturbing the S layers. It is instead, as Longa and de Jeu argue [59], entropically more favorable to disrupt the S layering by forming a RN phase. By extending McMillan’s theory of the N–S_A_ transition [60], Longa and de Jeu used a mean-field approach to solve for the one-particle distribution functions of monomers and dimers. Although Berker and collaborators [27,53,54,56] too considered dipolar interactions to be fundamental, they viewed the S phase as a percolating network of antiparallel pairs where frustration is avoided only because of liquid positional disorder. In our view, contrary to the conclusions of Figueirinhas *et al.* [17], it is difficult to exclude either theory in favor of the other, because the fundamental experimental predictions of the spin-gas theory, and of the McMillan-like theory are quite similar: They both predict a roughly *T*-independent value for the smectic layer thickness and antiparallel associated pairs.

It is worth mentioning other approaches to explain reentrancy in LC. For example, Luckhurst and Timini [61] found a N → S_A_ → RN phase sequence upon decreasing *T*. They only assumed a linear *T* dependence of the parameter *α* that in the original McMillan theory [60] is empirically related to the length of the molecular alkyl chain. In practice, as *α* decreases with decreasing *T*, the particles described by the theory turn from dimers to monomers. Ferrarini *et al.* [62] showed that reentrant phases can also be recovered by considering chemical reactions of isomerization and dimer formation under reasonable conditions.

All the models and theories discussed above refer to polar molecules. It it natural that most theoretical work has focused on this class of LC’s, because experimentally reentrancy is predominantly found in LC molecules with strong dipolar groups (typically cyano). However, RN phases have also been observed in nonpolar substances [29,30]. Dowell [63] devised a lattice theory for a single component system that focuses on molecular chain flexibility. In Dowell’s model the S phase is formed because of segregated packing of cores and tails. As *T* is lowered, however, the molecular chains become increasingly more stiff, destabilizing the S layers. Eventually, it becomes entropically more favorable to disrupt S layering such that a RN phase is formed. X-ray [57] and ESR [45] experiments support Dowell’s scenario of reentrancy in systems that do not show signs of dimerization [29,30]. Bose *et al.* [64] extended McMillan’s theory to include chain flexibility and also obtained a RN for a single component nonpolar system.

## 5. Computer Simulations

In modern times vast possibilities of theoretical investigation have been ushered by the growth of computational power and the sophistication of simulation algorithms. More and more complex fluids and microscopic structures can now be simulated [65]. Computer simulations were first applied to the study of RN’s by Netz and Berker [56]. They solved numerically the lattice spin-gas model introduced in Section 4 with Monte Carlo (MC) calculations. The first off-lattice MC simulations were performed only in 2005 by de Miguel and Martín del Río [66]. They considered a single-site molecular model for mesogens, where molecules are represented by parallel hard-core ellipsoids with a spherically symmetric square well potential (around each molecular long axis). In that study a RN phase and two tricritical points are found, one located on the N-S_A_ transition line and the second one on the S_A_-RN transition line. In this simple model, the presence of a RN in a nonpolar LC is rationalized in terms of entropy. In the S_A_ phase the energy is minimized and within the fluid state no further decrease is possible. However, the free energy can still be minimized by an increase in entropy. Thus, the loss of positional order is entropically driven [66].

Though the system of parallel hard-core ellipsoids [66] offers a clear picture of the origin of reentrancy in nematics, it has the shortcoming of neglecting rotational dynamics altogether, because the molecular long axes point in the same fixed direction. Suppressing orientational dynamics leaves open the question of whether the RN in such simple models is robust against orientational fluctuations.

### 5.1. Confined Mesogens

Confined fluids have become an extremely active field of research in recent years [67–69]. Novel phenomena take place in liquids confined to narrow spaces, e.g., wetting, layering and many others induced by the interaction with the confining surface. The interplay of these new phenomena with the physics of bulk fluids gives the possibility of both testing our understanding of molecular mechanisms, and devise concrete applications not otherwise feasible. In fact, applications of confined fluids range from nanotechnology to biomedical devices. It is therefore natural that LC properties and phase transitions come under a new scrutiny. Furthermore, nanoconfinement is a physically sensible (and realistic) way of inducing ordering fields into the fluid system.

Below, we review computer simulations of a pure LC system confined between walls separated by a nanoscopic distance (few tens of molecular diameters) performed by the present authors and their colleagues [12,13]. These are the first simulations to fully take into account a three dimensional fluid with rotational degrees of freedom.

#### 5.1.1. Model

Because the focus is on the dynamical behavior of the LC’s, molecular dynamics (MD) was employed. Specifically, we [12,13] adopted the Gay–Berne–Kihara (GBK) model for prolate mesogens [70]. The GBK model potential conveniently takes into account both the anisotropy of mesogen interaction and the spherocylindrical molecular shape [71] which is considered to approximate prolate mesogens better than ellipsoids [70].

In the GBK model the interaction between a pair of spherocylinders *i* and *j* depends on the molecular orientations represented by the unit vectors ***û****_i_* and ***û****_j_*, respectively and their distance ***r****_ij_* ≡ ***r****_i_* − ***r****_j_*. Specifically

(8) $$u_{\text{ff}}=4{ɛ}_{\text{ff}}\left({\hat{r}}_{ij},{\hat{u}}_{i},{\hat{u}}_{j}\right)\left[{\left(\frac{\sigma }{d_{ij}^{m}}\right)}^{12}-{\left(\frac{\sigma }{d_{ij}^{m}}\right)}^{6}\right]$$

where ***r̂*** ≡ ***r***/*r*, *r* ≡ |***r***|, and the function $d_{ij}^{m}(r_{ij},{\hat{u}}_{i},{\hat{u}}_{j})$ is the *minimum* distance between that pair of molecules [72]. The orientation-dependent interaction strength in Equation 8 is given by

(9) $$\begin{array}{l} {ɛ}_{\text{ff}}\left({\hat{r}}_{ij},{\hat{u}}_{i},{\hat{u}}_{j}\right)=\in_{\text{ff}}{\left\{1-\frac{\chi \prime }{2}\left[\frac{{\left({\hat{r}}_{ij}\cdot {\hat{u}}_{i}+{\hat{r}}_{ij}\cdot {\hat{u}}_{j}\right)}^{2}}{1+\chi \prime {\hat{u}}_{i}\cdot {\hat{u}}_{j}}+\frac{{\left({\hat{r}}_{ij}\cdot {\hat{u}}_{i}+{\hat{r}}_{ij}\cdot {\hat{u}}_{j}\right)}^{2}}{1-\chi \prime {\hat{u}}_{i}\cdot {\hat{u}}_{j}}\right]\right\}}^{2}\\ \;\;\;\;\;\;\;\;\;\;\;\;\;\;\;\;\;\;\;\;\;\;\;\;\;\;\;\;\;\;\;\;\;\;\times \frac{1}{\sqrt{1-\chi^{2}{\left({\hat{u}}_{i}\cdot {\hat{u}}_{j}\right)}^{2}}} \end{array}$$

where the parameters *χ* and *χ*′ are given by

(10a) $$\chi \equiv \frac{\kappa^{2}-1}{\kappa^{2}+1}$$

(10b) $$\chi \prime \equiv \frac{\sqrt{\kappa \prime }-1}{\sqrt{\kappa \prime }+1}$$

In these last two expressions parameters *κ* = *L* + 1 (*L* in units of *σ*), and *κ*′ represents the interaction strength for a side-side relative to an end-end configuration of a pair of spherocylinders.

We consider a LC system confined along the *z* direction by two planar, structureless walls. We model the fluid-substrate interaction via

(11) $$u_{\text{fs}}=4\in_{\text{fs}}\rho_{s}\left[{\left(\frac{\sigma }{d_{ik}^{m}}\right)}^{10}-{\left(\frac{\sigma }{d_{ik}^{m}}\right)}^{4}g\left({\hat{u}}_{i}\right)\right]$$

where the parameter *ε*_fs_ is the strength of fluid-substrate interaction and *ρ*_s_ = 2/*ℓ*^2^ is the areal density of the substrate where $\ell /\sigma =1/\sqrt[{3}]{4}$ is the lattice constant of a single layer of atoms arranged according to the (100) plane of the face-centered cubic lattice [12]. The diameter *σ* of these substrate atoms is taken to be the same as the diameter of a spherocylinder of the confined fluid phase.

As already mentioned, a solid substrate is also a physically realistic way of inducing an ordering field in a LC system. A number of experimental techniques were devised to realize specific substrate features, and especially anchoring. Some example are rubbing, polishing, exposing to photolithographic relief, or evaporation of oxide films [73]. In Equation 11, 0 ≤ g(*û*) ≤ 1 is the so-called “anchoring function”. It allows one to discriminate energetically different orientations of a molecule with respect to the substrate plane. We employed degenerate *planar* anchoring [74]

(12) $$g(\hat{u})={\left(\hat{u}\cdot {\hat{e}}_{x}\right)}^{2}+{\left(\hat{u}\cdot {\hat{e}}_{y}\right)}^{2}$$

where *ê_α_* (*α* = *x*, *y*, *z*) is a unit vector pointing along the *α*-axis of a space-fixed Cartesian coordinate system. Hence, any molecular arrangement parallel with the substrate plane is energetically favored, whereas a homeotropic alignment of a molecule (*û* ‖ *ê*_z_) receives an energy penalty by “switching off” the fluid-substrate attraction altogether.

The MD simulations were carried out using a velocity Verlet algorithm for linear molecules [75]. After equilibrating the system to the chosen pressure and *T*, the fluid system is simulated in the microcanonical ensemble to obtain information on its dynamics [13]. We simulated systems containing up to *N* = 1500 molecules. Quantities of interest will be expressed in the customary dimensionless (*i.e.*, “reduced”) units. For example, length will be expressed in units of *σ*, energy in units of *ε*_ff_, temperature in units of *ε*_ff_/*k*_B_, time in units of (*σ*^2^*m*/*ε*_ff_)^1/2^ using *m* = 1, and pressure *P*‖ in units of *k*_B_*T*/*σ*^3^ where $P_{∥}=\frac{1}{2}(P_{\text{xx}}+P_{\text{yy}})$ is related to diagonal components of the pressure tensor **P** acting in the *x* − *y* plane.

#### 5.1.2. Results

To characterize the structure of N, S_A_, and RN phases a suitable order parameter is the so-called alignment tensor defined as [76,77]

(13) $$Q\equiv \frac{1}{2N}\sum_{i=1}^{N}\left(3{\hat{u}}_{i}⊗{\hat{u}}_{i}-1\right)$$

where “⊗” denotes the direct (*i.e.*, dyadic) product and 1 is the unit tensor. Hence, **Q** is a real, symmetric, and traceless second-rank tensor which can be represented by a 3 × 3 matrix. Its largest eigenvalue is commonly used as the N order parameter *S*.

To characterize the layering of the fluid in the direction of *n̂* characteristic of S phases, a suitable order parameter is given by the leading coefficient of the Fourier series of the local density *ρ*(***r***)

(14) $$\Lambda \equiv \frac{1}{N}\langle \left|\sum_{i=1}^{N}\text{exp}\left[\frac{2\pi i\left(r_{i}\cdot \hat{n}\right)}{d}\right]\right|\rangle$$

where *d* is the spacing between adjacent S_A_ layers. From Equation 14 it is also apparent that Λ ∈ [0, 1], where Λ ≈ 0, and Λ ≈ 1 in ideal N and S_A_ phases, respectively.

**Figure 3.**
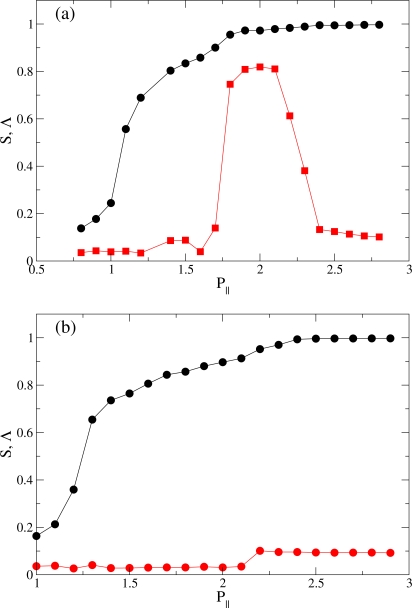
Plots of nematic *S* ( 
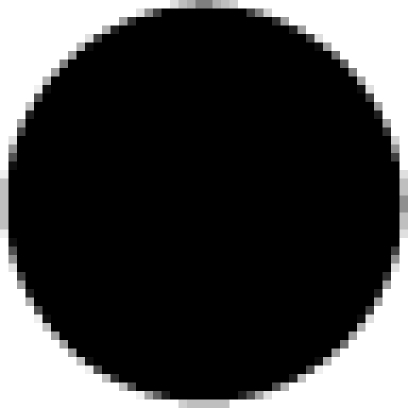
) and smectic order parameter Λ ( 
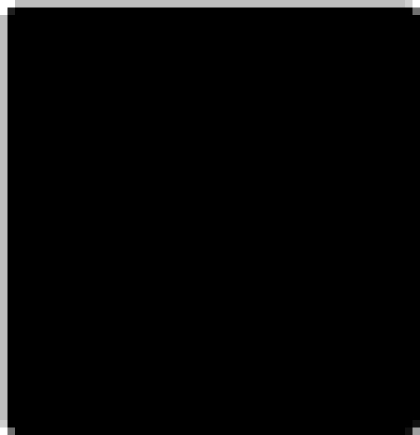
) as functions of pressure *P*_‖_ at (**a**) *T* = 4.0; and (**b**) at *T* = 6.0.

Plots of the dependence of *S* and Λ on *P*_‖_ are presented in Figure 3 for *T* = 4.0 and 6.0. Both *S* and Λ are small at low pressures characteristic of the I phase. Here we adopt a value *S* ≃ 0.4 as a heuristic threshold for the N phase [78,79]. From Figure 3 it therefore appears that the N phase forms somewhere above *P*_‖_ ≃ 1.0 (*T* = 4.0) and 1.2 (*T* = 6.0), respectively.

**Figure 4.**
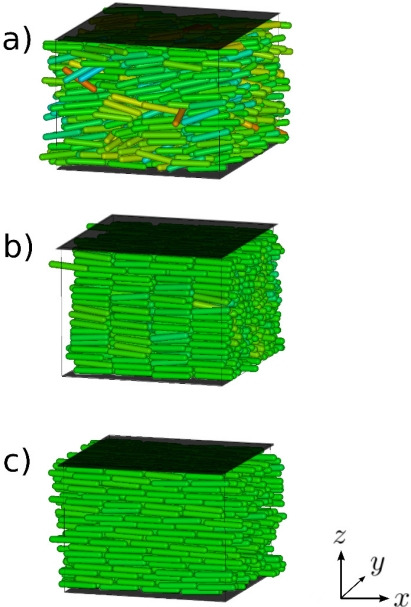
“Snapshots” of characteristic configurations at *T* = 4.0; (**a**) *P*_‖_ = 1.6 (N); (**b**) *P*_‖_ = 2.1 (S_A_ ); (**c**) *P*_‖_ = 2.9 (RN) (see Figure 3). Molecules are aligned with *n̂* parallel to the *X* axis, whereas flat surfaces on top and bottom in dark represent the solid substrates.

At *T* = 4.0 there is a clear sign of a RN phase as *S* increases and Λ reaches a maximum value and then drops to small values for *P*_‖_ *≃* 2.3; thus, a sequence of phases I-N-S_A_-RN is found at *T* = 4.0 as *P* increases. At *T* = 6.0, Λ never rises above the residual value of about 0.1. Hence, the S_A_ phase does not form at this *T*. Nevertheless, we notice a small, step-like increase at *P*_‖_ *≃* 2.2 in the plot of Λ. At this and all larger pressures considered the nematic order is high and increases even further reflected by a monotonic increase of *S* toward its limiting value 1.0. For *P*_‖_ ≳ 2.2 the confined fluid exhibits structural features of the RN phase (see [13]). Three characteristic snapshots of the system are shown in Figure 4. The increasing orientational order of the system as *P*_‖_ increases is apparent. Incidentally, we note that the seemingly value *S* = 1 in the RN phase is a finite-size effect, as was demonstrated by Eppenga and Frenkel [80].

**Figure 5.**
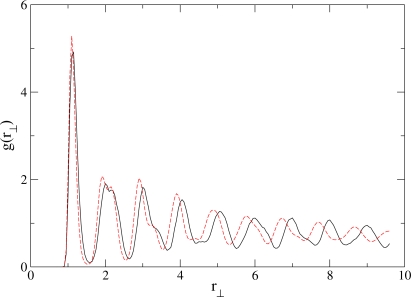
Perpendicular radial distribution function for a bulk (solid line) and confined (dashed line) simulation. Both states are in the RN phase at *T* = 4.0.

A question naturally arises: What is the relation between the RN phase found in this simulated confined fluid and the bulk RN’s? Our simulations show that the phase diagram of the confined system is qualitative similar to phase diagram of the bulk fluid. The only change induced by the confining surfaces is to lower the pressure of the phase transitions with respect to the bulk system. This shift in the phase diagram is a well-documented confinement effect. Here, we consider spatial correlations between the centers of mass of molecules in the direction ***r***_⊥_ perpendicular to the orientation of a reference molecule, where ***r***_⊥_ ≡ ***r*** − (***û*** · ***r***)***û.*** The perpendicular radial distribution function *g*(*r*_⊥_) is the probability to find a molecule at distance *r*_⊥_ = |***r***_⊥_| from a reference molecule. In Figure 5 we compare *g*(*r*_⊥_) for a bulk and a confined system in the RN phase. It is apparent that the maxima of *g*(*r*_⊥_) for the confined system are shifted to lower values of *r*_⊥_ with respect to the bulk case. This shift reflects the substrate-assisted efficient packing of molecules and therefore explains the shift of the S_A_-RN transition to lower *P*_‖_ than in the bulk. However, the structure of the confined and bulk RN phases are very similar.

Next, we address the important question of dynamics in the RN phase. To this end, we considered a *specialized* mean square displacement (MSD) to calculate displacements of molecules in the direction of their long axes. Specifically, we define $r_{i}^{∥}\equiv {\hat{u}}_{i}\cdot r_{i}$ and the associated MSD in the direction of the molecular long axes

(15) $$\langle \Delta r_{∥}^{2}{\left(\tau \right)\rangle }_{t}\equiv \;\frac{1}{N}{\langle \sum_{i=1}^{N}{\left[r_{i}^{∥}(t+\tau )-r_{i}^{∥}(t)\right]}^{2}\rangle }_{t}$$

From Equation 15 a diffusion coefficient can be extracted using an Einstein relation, namely

(16) $$D_{∥}=\underset{\tau \to \infty }{\text{lim}}\frac{1}{2\tau }\langle \Delta r_{∥}^{2}{\left(\tau \right)\rangle }_{t}$$

**Figure 6.**
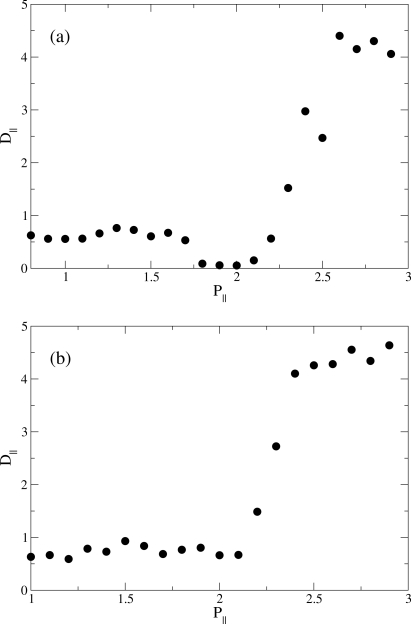
Parallel self-diffusion coefficient *D*_‖_ as a function of transverse pressure *P*_‖_. (**a**) Calculations at *T* = 4.0; (**b**) Calculations at *T* = 6.0.

Figure 6 shows a dramatic increase of *D*_‖_ as one enters the RN phase whereas lower-pressure (I, N, or S_A_) phases exhibit rather small self-diffusivity. In particular, the S_A_ phase is characterized by nearly vanishing self-diffusion constants which can be rationalized as above where we argued that the relatively compact layered structure makes it difficult for molecules to diffuse out of their original layer and penetrate into a neighboring one. The dramatic increase in mass transport in the direction of *n̂* in combination with nearly perfect nematic order prompted us to refer to liquid crystals in the RN phase as “supernematics” [12].

There are, in fact, experimental observations that are consistent with our simulations. For example, by extracting longitudinal relaxation rates from data of Miyajima *et al.* [32], one finds that in the RN phase these relaxation rates are considerably lower than those characteristic of the N phase. At the same *T*, relaxation rates can be converted into correlation times that are significantly shorter in the RN as opposed to the N phase. However, this remains speculative, until experiments prove that translation diffusion is the main relaxation mechanism at this *T*. Stronger evidence is found in the conductivity experiments performed by Ratna [16] (see Section 3).

One may ask: Why is the diffusivity so large in the direction of the long molecular axis? As interesting analogy can be drawn with the case of diffusion in systems such as zeolites or single-wall carbon nanotubes. When the diameter of a diffusing particle matches the size of the confining nanochannel then the diffusivity increases markedly. This effect is known as “levitation” [14,15]. In our case, the high degree of orientational order in the RN phase causes a molecule to be trapped within the “cage” formed by its first-neighbors which, because of the prolate geometry, is effectively a narrow channel. Furthermore, the attractive intermolecular forces are overcome because the fluid is in a dense state (either low *T* or high pressure). Thus, within this channel a molecule fits the condition of the levitation effect.

## 6. Conclusions

In this work, we have reviewed experimental and theoretical investigations of structural and dynamical properties of RN phases. It is surprising that after almost 40 years of research many details of RN phases are still escaping a comprehensive understanding. The main difficulty is the variety of substances exhibiting RN’s. Pure substances and mixtures alike have been found with a RN in their phase diagrams. Polar and nonpolar molecules exhibit reentrancy alike. It is clear to the present authors that more experiments are necessary to settle some important, yet still open questions. For example: (i) How does the nematic order change upon crossing the S_A_-RN transition? (ii) Is frustration really the mechanism underlying the reentrant behavior in LC’s with a bilayer S phase? (iii) Experimentally, what is the mechanism driving reentrancy in LC’s that do not form dimers? (iv) Do RN phases of single-component, nonpolar molecules really exhibit the huge self-diffusivity in the direction of *n̂* predicted by our simulations [12,13]?

Regarding the dynamical behavior, it is at present safe to assume that though many LC systems have a RN phase, their dynamics need not be alike in any respect. The reason is that different microscopic mechanisms may be responsible for reentrancy in different LC systems depending on the details of the intermolecular interaction potential. At the time of writing some experimental evidence exists [16] suggesting that last question may indeed have a positive answer. Moreover, our findings [12,13] seem consistent with an extrapolation of longitudinal relaxation rates of NMR experiments [32].
